# Liver Transplantation for Intrahepatic Cholangiocarcinoma: What Are New Insights and What Should We Follow?

**DOI:** 10.3389/fonc.2021.841694

**Published:** 2022-01-21

**Authors:** Dawei Sun, Guoyue Lv, Jiahong Dong

**Affiliations:** ^1^ Department of Hepatobiliary and Pancreatic Surgery, The First Hospital of Jilin University, Changchun, China; ^2^ Hepato-Pancreato-Biliary Center, Beijing Tsinghua Changgung Hospital, Tsinghua University, Beijing, China

**Keywords:** intrahepatic cholangiocarcinoma (iCCA), liver transplantation (LT), transplant outcome, tumor burden, tumor biology, pretransplant bridging

## Abstract

Intrahepatic cholangiocarcinoma (iCCA) is a complex malignancy carrying poor prognosis. Liver transplantation (LT) was historically contraindicated for iCCA, due to poor outcomes after LT. However, an increasing number of studies have challenged this premise, because LT alone or combined with neoadjuvant chemotherapy has achieved relatively satisfactory transplant outcomes in well selected iCCA cases. This current review based on existing clinical researches, evinced that LT might serve as a viable option in iCCA cases as follows: ① unresectable tumor restricted to 2 cm, along with context of chronic liver diseases; and ② unresectable tumor locally advanced within the liver (without extrahepatic metastasis or vascular invasion) but responses to tumor down-staging treatments (namely, systemic neoadjuvant therapy and/or locoregional therapy). On the contrary, it is recommended as contraindications in iCCA cases as follows: ① patients with tumor progression while waiting for a transplant (increase of diameter, macrovascular invasion, new nodules, escalation of carbohydrate antigen 19-9, or extrahepatic spread); ② patients with iCCA recurrence. Conclusively, tumor burden, tumor biology, and response to down-staging strategies should be taken into consideration before LT. Whereas, the concept of “locally advanced stage” remains to be defined in the future, especially the optimized combination of “maximum size of largest lesion”, “number of lesions”, with/without “tumor differentiation”, just like the Milan criteria which is widely used for hepatocellular carcinoma. Given the scarcity of donor organ, and also the debate about LT in iCCA, accurate consensus about LT for iCCA patients is still urgently warranted.

## Introduction

Intrahepatic cholangiocarcinoma (iCCA), a fatal malignancy, arises from the intrahepatic bile ducts proximal to the second-order division (1). As reported in GLOBOCAN Cancer Statistics 2020, iCCA accounts for 10–15% of primary liver cancer cases, with more than 90,567 new cases and 83,018 deaths that occurred in 2020 worldwide (2). Unluckily, iCCA is usually tough to diagnose and treat because of its disease complexity, and its incidence rate is increasing steadily (3–5). Even worse, many cases are diagnosed in advanced stages, because this disease is usually asymptomatic in its early stage (3). Although surgical resection offers the best possibility of cure for patients with localized stage, it does not achieve satisfactory outcomes due to high postoperative recurrence rate (6, 7). Consequently, the prognosis of iCCA patients is usually dismal, with a 5-year survival rate after resection ranging from 25 to 40% (5).

Notably, for iCCA cases in context of advanced cirrhosis, liver transplantation (LT) possesses the advantages of achieving negative oncologic margin, eliminating intrahepatic micrometastases, and also solving the underlying liver disease. However, the presence of iCCA was once considered as a contraindication of LT because of dismal transplant outcomes, with 1- and 3-year overall survival (OS) rates of only 19.4–38% and 4.9–10%, respectively (8, 9). However, increasing number of studies have challenged this conservative recognition, due to significantly improved transplant outcomes that have been achieved for iCCA patients (1-, 3-year OS rates of 83.3–100% and 47.91–83.3%, respectively) (10, 11). The possible reasons accounting for previously dismal prognosis, might be due to inappropriate patient selection and neoadjuvant therapeutic absence (5).

To our knowledge, the consensus used for LT in iCCA cases has not been reached yet. Therefore, we conducted this current review according to available pieces of literature, aiming at providing better evidence for clinical work. Herein, this review mainly focuses on transplant outcomes (actuarial survival and tumor recurrence), tumor burden (diameter and number), tumor biology (tumor differentiation and vascular invasion), and pretransplant bridging (neoadjuvant therapy and pre-transplant locoregional therapy) exclusively for iCCA, rather than other biliary malignancies.

## Transplant Outcomes

Using the keywords “*liver transplantation*” and “*intrahepatic cholangiocarcinoma*” in the database of Pubmed, we manually selected clinical articles which investigated LT in iCCA cases (8, 9, 12–17). During this process, our main interests were survival outcomes, including OS and/or recurrence-free survival (RFS). Here, we summarized the study characteristics and the transplant outcomes in **Table 1**. According to the timeline, we categorized the outcomes of LT in iCCA into three phases. The first stage ranging from 1988 to 2001, had prognostic outcomes that were extremely poor, probably due to undefined patient selection, and also the absence of effective pretransplant treatment modalities (8, 9, 12). The second stage ranging from 2011 to 2016, had prognostic outcomes that improved gradually, due to the relatively defined patient selection (“*very early stage” and/or “locally advanced stage”*), and also locoregional therapy involvement (10, 13–16). The third stage ranging from 2018 to 2021, had LT achieved the most favorable outcomes despite iCCA cases of advanced stages, due to the application of neoadjuvant chemotherapy (11, 17). Admittedly, potential confounding factors, namely, tumor burden, tumor biology, and pretransplant bridging, should have affected the transplant outcomes. Therefore, we analyze these three factors one by one after this part.

**Table 1:** Characteristics of identified clinical studies investigating LT in iCCA cases.

1st Author [Ref.]	Year	Country	No. of cases	Pathological features (*No. of cases*)	Pretransplant treatments *(No. of cases)*	Actuarial survival (OS rate)	Tumor recurrence (RFS rate)	Follow-up time (months)
O’Grady et al. (8)	1988	UK	13	Not available	Not available	38.4% for 1 year 10% for 3 year 0% for 5 year	Not available	Not available
Pichlmayr et al. (9)	1997	Germany	24	Not available	Not available	19.4% for 1 year 4.9% for 3 year 0% for 5 year	Not available	Not available
Shimoda et al. (12)	2001	USA	16	pTNM stage I/II/III/IV (2/2/3/9), >2 lesions (12), vascular invasion (3), lymph node metastasis (2)	Not available	62% for 1 year 39% for 3 year	70% for 1 year 35% for 3 year	Not available
Hu et al. (13)	2011	China	20	pTNM stage I/II/III (4/4/12), ≥2 lesions (11), macrovascular invasion (12), microvascular invasion (16), lymph node metastasis (9), poor differentiation (11)	Neoadjuvant therapy: without definite scheme or patient distribution	84.2% for 1 year 32.7% for 3 year 21.8% for 5 year	55.6% for 1 year 28.8% for 3 year 18.8% for 5 year	Median 15.0 (2–96)
Vallin et al. (14)	2013	France	4	*Advanced stage* (4), vascular invasion (1)	Not available	75%% for 1 year 75% for 3 year	75%% for 1 year 75%for 3 year	8-52
Sapisochin et al. (15)	2014	Spain	29	*Advanced stage* (21), macrovascular invasion (2), microvascular invasion (3)	Locoregional therapy: TACE (8), RFA (3), PEI (2)	79% for 1 year 61% for 3 year 45% for 5 year	89% for 1 year 71% for 3 year 71% for 5 year	Median 36.4 (1.8–117.8)
Takahashi et al. (16)	2016	USA	13	Tumor size ranging from 1.0 to 3.3 cm in diameter, vascular invasion (1), poor differentiation (0), lymph node metastasis (1)	Locoregional therapy: TACE (4), RFA (1)	Not available	67% for 1 year 42% for 3 year	Median 18.8
Sapisochin et al. (10)	2016	International cooperation	48	*Advanced stage* (33), macrovascular invasion (2), microvascular invasion (11), poor differentiation (6)	Locoregional therapy: TACE (12), ablation (8), TACE +ablation (3)	83.3% for 1 year 47.9% for 3 year 31.3% for 5 year	75% for 1 year 41.7% for 3 year 27.1% for 5 year	Median 57.3 (23.4–104.5)
Lunsford et al. (11)	2018	USA	6	*Advanced stage* (6), macrovascular invasion (0), microvascular invasion (2), lymph node metastasis (2), poor differentiation (3)	Neoadjuvant chemotherapy: platinum-based therapy and gemcitabine (6)	100% for 1 year 83.3% for 3 year 83.3% for 5 year	50% for 1 year 50% for 3 year 50% for 5 year	Median 36 (29–51)
McMillan et al. (17)	2021	USA	18	*Advanced stage* (18), lymphovascular invasion (6)	Neoadjuvant chemotherapy: cisplatin/gemcitabine (18)	100% for 1 year 71% for 3 year 57% for 5 year	70% for 1 year 52% for 3 year	Median 26

“Advanced stage”, single lesion >2 cm or multiple lesions; TACE, transarterial chemoembolization; RFA, radiofrequency ablation; PEI, percutaneous ethanol injection.

## Tumor Burden

As reported, tumor burden, namely, tumor size and number, are known factors correlated with outcomes in iCCA patients receiving surgery (7, 18, 19). Similarly, tumor burden is also tightly associated with post-LT outcomes for iCCA patients. Here, we emphasized this correlation by timeline.

In 2011, the retrospective single-center study enrolling 20 iCCA cases from China, revealed that multiple lesions (n ≥2) significantly correlated with poor OS [*risk ratio (RR) = 10.695, p = 0.026 via univariate analysis; RR = 24.150, p = 0.024, via multivariate analysis*] and poor RFS (*RR = 11.524, p = 0.022 via univariate analysis; RR = 9.118, p = 0.047, via multivariate analysis*), when compared with single lesion (13). In 2014, the retrospective multicenter cohort study enrolling 29 iCCA cases from Spain, demonstrated that iCCA patients restricted to single tumor ≤2 cm (*very early stage*) acquired better 1-, 3-, 5-year actuarial survival rates, when compared with those involving single tumor >2 cm or multinodular tumors (*advanced stage*) (*100%, 73%, 73% vs. 71%, 43%, 34%, respectively*) (15). Meanwhile, this study also showed that iCCA patients in “*very early stage*” group presented lower 5-year recurrence rate when compared to those in “*advanced stage*” group (*0% vs. 36.4%, p = 0.02*), and the hazard ratio (HR) for tumor recurrence increased with the increasing of tumor size dividing line. In 2016, the international retrospective study of 48 iCCA cases further evinced that patients in “*very early stage*” group (15 cases) gained better prognosis when compared with those in “*advanced stage*” group (33 cases), with the condition that no significant differences existed in preoperative characteristics between groups, in terms of 1-, 3-, and 5-year actuarial survival rates (*93%, 84%, 65% vs. 79%, 50%, 45%; p = 0.02*) and 1-, 3-, and 5-year cumulative recurrence rates (*7%, 18%, 18% vs. 30%, 47%, 61%; p = 0.02*) (10). In 2021, it is suggested that only iCCA cases in the setting of liver cirrhosis, with early stage (single tumor ≤2 cm without vascular invasion) would be acceptable for LT, according to consensus statement and recommendation by the Spanish Society of LT (20). The latest research in 2021, one meta-analysis based on 18 studies enrolling 355 cases and a registry study of 385 cases, demonstrated iCCA patients with “*advanced stage*” exhibiting an inferior pooled post-transplant 5-year RFS rate (*34%, 95%CI:23–46%*), when compared with patients with “*very early stage*” (*67%, 95%CI:47–86%*) (21). Importantly, this meta-analysis included some different studies, which were based on other types of biliary malignancies, namely, combined hepatocellular-cholangiocarcinoma (cHCC-CCA) alone or mixed with iCCA. For these types of biliary malignancies, they possess relatively different prognosis from iCCA due to their biological features.

## Tumor Biology

Tumor biology is closely correlated with cancer progression. Thus, it needs to be cautious when choosing LT for aggressive iCCA cases. Here, we also delineated by timeline in terms of tumor differentiation and vascular invasion as following.

As one characteristic of tumor aggressiveness, tumor differentiation was reported to be associated with transplant outcomes in iCCA. In 2016, Takahashi et al. from the USA performed a retrospective study enrolling 13 iCCA cases, among which 4 cases were diagnosed as well-differentiated and 9 cases were diagnosed as moderately-differentiated. After following more than 3 years, no recurrence occurred in the group with well-differentiated tumor, but 7 cases (78%) suffered from tumor recurrence in the moderately-differentiated group (*median RFS of 13.0 months, and with the 1- and 3-year RFS rates of 56 and 22%, respectively*) (16). In 2016, the international retrospective study performed by Sapisochin et al. further revealed that poor tumor differentiation significantly correlated with tumor recurrence not only *via* univariate analysis (*HR = 3.8, 95%CI:1.3–11.2, p = 0.01*) but also *via* multivariate analysis (*HR = 6.1, 95%CI:1.9–20.2, p =0.003*) (10).

Being another characteristic indicating tumor aggressiveness, vascular invasion was also reported to be associated with transplant outcomes in iCCA. In 2013, Valli et al. retrospectively analyzed 4 iCCA cases, with the follow-up interval ranging from 8 to 52 months, and they found that tumor relapse was found in 100% of cases with vascular invasion (1/1) compared with 0% of those without vascular invasion (0/3) (14). In 2016, the international retrospective study conducted by Sapisochin et al., also emphasized that microvascular invasion was significantly correlated with tumor recurrence not only *via* univariate analysis (*HR = 3.5, 95%CI:1.4–8.5, p = 0.006*) but also *via* multivariate analysis (*HR = 4.7, 95%CI:1.6–13.8, p = 0.005*) (10).

Similarly, the 2021 consensus statement and recommendation by the Spanish Society of LT suggested that it is motivated to exclude it from the waiting list, in case of tumor progression (increase of diameter ≥2 cm, vascular invasion, new nodules, obvious escalation of carbohydrate antigen 19-9, or extrahepatic spread). Moreover, it also raised that retransplantation was contraindicated for recurrent iCCA cases in this consensus statement and recommendation (20).

## Pretransplant Bridging

As modalities of pretransplant bridging, neoadjuvant therapy and pre-transplant locoregional therapy contribute to tumor downstaging. However, only few studies investigated the role of downstaging therapy exclusively in iCCA patients. On the contrary, most of the studies were based on patients diagnosed with combined cHCC-CCA alone or mixed with iCCA.

Neoadjuvant therapy gradually becomes promising for iCCA treatment, due to its critical role of controlling disease progression, and also the possibility of converting unresectable cases to LT. In 2011, Hong et al. collected 38 cholangiocarcinoma cases (namely, intrahepatic and hilar cases) receiving LT, and their results firstly expounded that neoadjuvant and adjuvant therapies after LT resulted in a better 5-year OS rate compared with adjuvant therapy only or no therapy (*47, 33, and 20%, respectively; p = 0.03*) (22). In details, the neoadjuvant therapy protocol consisted of transarterial chemoembolization (TACE) or stereotactic radiotherapy, along with chemotherapy (capecitabine or 5-fluorouracil based regimen). In 2018, one prospective case-series including 6 unresectable iCCA patients with locally advanced stage (*maximum tumor size of largest lesion ≥3.5 cm, cumulative tumor diameter ranging from 8.1 to 17.9 cm, number of lesions ranging from 2 to 10; without extrahepatic metastasis or vascular invasion*) were treated with neoadjuvant therapy (*gemcitabine-based regimen, and also radiotherapy*) while awaiting LT, subsequently demonstrated satisfied transplant outcomes, with the 1-, 3-, and 5-year OS rates of 100, 83.3, and 83.3%, and also 50% RFS rate at 1, 3, and 5 years (11). In 2021, another study assessed the transplant outcomes in 18 unresectable iCCA cases with locally-advanced stage (*cumulative tumor diameter ranging from 8.1 to 17.9 cm, number of lesions ranging from 1 to 10*), who received neoadjuvant therapy (*gemcitabine-based regimen*) and exhibited disease control for six months before LT (17). This contemporary study showed that these selected iCCA patients receiving LT had relatively satisfied 1-, 3-, 5-year OS rates of 100, 71, and 57%, and also relatively satisfied 1- and 3-year RFS rates of 70 and 52%.

Meanwhile, pretransplant loco-regional therapy also plays a vital role for HCC and cHCC-CCA, since significantly improved transplant outcomes have been achieved for responders (23, 24). More specifically, Antwi et al. investigated the role of pre-transplant loco-regional therapy in 19 cHCC-CCA patients, and their results demonstrated that cHCC-CCA responders acquired superior 3-year OS rate (*92% vs. 23%, p = 0.03*), but identical RFS rate (*log rank p = 0.6452*), when compared with those non-responders (24). However, it still needs to be testified whether pre-transplant loco-regional therapy will also generate comparative effect when used in iCCA patients.

## Discussion

Up until now, the consensus used for LT in iCCA (or cHCC-CCA) patients has not been reached yet, but the Milan criteria widely used for LT in HCC has guided clinical work for decades. During the past decades, transplant surgeons have continuously managed to optimize two objectives about iCCA—tackling more cases of this extremely troublesome disease of unresectable stage (also termed as quantity), and also trying to gain relatively satisfactory transplant outcomes of patients (also termed as quality).

According to our literature review results, the transplant outcomes were relatively satisfied in iCCA cases of “*very early stage*” (10, 15), and also in iCCA cases of “*advanced stage*” response to pretransplant treatments (namely, neoadjuvant chemotherapy and/or locoregional therapy) (11, 17). But, it portended relatively poor outcomes in cases with vascular invasion, poor tumor differentiation, or “*advanced stage*” without pretransplant treatments (10, 14–16, 21). Indeed, many other researchers also have tried to exploit this issue based on patients with cHCC-iCCA or cHCC-iCCA along with iCCA, except for those insights exclusively for iCCA as mentioned before, since the number of iCCA cases conducted in each individual transplant center is relatively limited.

Herein, some emerging insights regarding LT in cHCC-iCCA or cHCC-iCCA along with iCCA, are also listed by timeline. In 2015, Facciuto et al. collected 32 patients (either iCCA or cHCC-CCA) receiving LT, and their results showed that cHCC-iCCA or iCCA patients restricted to the Milan criteria achieved comparable prognosis when compared with matched HCC patients in control group (64 cases), in terms of 5-year OS rate (*78% vs. 79%, p = 0.61*) but slightly high recurrence rate (*10% vs. 5%, p = 0.6*). Whereas, patients outside of the Milan criteria acquired inferior transplant prognosis when compared with patients within Milan, in terms of 5-year OS rate (*48% vs. 78%, p = 0.1*) and 5-year DFS rate (*32% vs. 78%, p = 0.04*) (25). In 2018, Lee et al. compared the transplant outcomes between iCCA/cHCC-CCA patients with early stage (diameter restricted to 2 cm) (12 cases) and HCC patients within the Milan criteria (319 cases), and their results also showed that those early staged iCCA/cHCC-CCA patients gained comparative 1- and 5-year survival rates with those matched HCC patients (*63.6%, 63.6% vs. 90.0%, 70.3%, respectively; P-value for log-rank of 0.25*), but iCCA/cHCC-CCA patients suffered from significantly higher cumulative recurrence risk (*33.3% vs. 11%, P-value for log-rank of 0.01*) (26). In 2020, De Martin et al. conducted a contemporary study enrolling 75 iCCA/cHCC-CCA patients (with diameter restricted to 5 cm) with median follow up of 25 (0–151) months, among which 49 cases received LT and 26 patients received liver resection (27). First, this study demonstrated that LT achieved better prognostic outcomes when compared to liver resection, in terms of improved 1-, 3-, and 5-year RFS rates (*87%, 79%, 75% vs. 69%, 45%, 36%; respectively, p = 0.004*) but identical 1-, 3-, and 5-year OS rates (*90%, 76%, 67% vs. 92%, 59%, 40%; respectively, p = 0.165*). Second, this study revealed that patients with tumor diameter ≤2 cm shared comparable transplant outcomes with patients with larger tumor size (ranging from 2 to 5 cm), in terms of 1-, 3-, and 5-year OS rates (*92%, 87%, 69% vs. 87%, 65%, 65%; respectively, p = 0.4*) and also comparable DFS rates (*p-value for log-rank of 0.43*). Third, multivariate analysis of this study evinced that it was tumor differentiation rather than tumor diameter that correlated with transplant outcomes. Accordingly, the authors of this study recommended LT for patients with unresectable iCCA/cHCC-CCA due to cirrhosis, under the criteria of tumor diameter less than 5 cm. Generally, this study is well-designed and meaningful, which not only challenges the conservative conception that iCCA was not suitable for LT, but also extends the tumor diameter from ‘*restricted to 2 cm*’ to ‘*restricted to 5 cm*’ for LT in iCCA/cHCC-CCA. Collectively, patient selection strategy (“*within Milan criteria”* or “*restricted to 5 cm*”) is significantly correlated with transplant outcomes in iCCA/cHCC-CCA based on these three emerging studies.

Based on the acquired evidence above, tumor burden, tumor biology, and pretransplant bridging modality should be taken into primary consideration when assessing transplant strategy in iCCA cases. LT might serve as a viable option in cases as follows: (I) unresectable tumor restricted to 2 cm, whilst arisen in context of chronic liver diseases; (II) unresectable tumor locally advanced within the liver (single lesion more than 2 cm, or number of lesions ≥2; without extrahepatic metastasis or macrovascular invasion), but responses to tumor down-staging modality (namely, systemic neoadjuvant therapy and/or loco-regional therapy). The items are summarized as above, which were consistent with the insights raised by other researchers (5, 20, 28, 29). Here, the optimized combination of “maximum size of single lesion” with/without “number of lesions” remained undefined, when compared with the Milan criteria. Neoadjuvant therapy includes gemcitabine based treatment, and locoregional treatment includes radiotherapy, TACE, and radiofrequency ablation (RFA). On the contrary, it is recommended as contraindication of LT in cases as follows: (I) patients suffering from tumor progression (increase of diameter, macrovascular invasion, new nodules, escalation of carbohydrate antigen 19-9, or extrahepatic spread) while waiting for a transplant; (II) patients diagnosed as recurrent iCCA (20). In order to present these above items more clearly, we summarize these items in **Table 2**.

**Table 2:** Summarized new insights of LT for iCCA cases according to literature review.

Indications	
I	Patients with unresectable tumor restricted to 2 cm, whilst arisen in context of chronic liver diseases (10, 15, 20, 29).
II	Patients with unresectable tumor locally advanced within the liver (single lesion more than 2 cm, or number of lesions ≥2), without extrahepatic metastasis or macrovascular invasion, but with responses to tumor downstaging (namely, systemic neoadjuvant therapy and/or loco-regional therapy) (5, 11, 17, 29). Whereas, the optimized combination of “maximum size of single lesion” with/without “number of lesions” remained undefined.
**Contraindications**	
I	Patients suffering from tumor progression waiting for a transplant (increase of diameter, vascular invasion, new nodules, escalation of carbohydrate antigen 19-9, or extrahepatic spread) (17, 20).
II	Patients diagnosed with recurrent iCCA (20).

When compared with conservative conception or “*very early stage*” selection standard, these well-selected protocols are expected to make more iCCA cases benefit from LT. Then, strategies to identify the locally advanced status of iCCA are extremely important, too. This correct diagnosis should depend on comprehensive imaging techniques, since there are limitations and advantages of imaging modalities used for iCCA diagnosis (30). For instance, magnetic resonance imaging (MRI) mainly showed the primary mass with enhanced assessment, computer tomography (CT) possessed the superiority of vascular invasion detection, and [^18^F]-fluorodeoxyglucose-positron emission tomography (18-FDG) PET contributes to accurate tumor staging by detecting metastasis reliably (namely, regional lymph node and distant metastasis) (1). However, sometimes iCCA is difficult to diagnose before LT despite using above imaging modalities. In this case, pretransplant biopsy remains the mainstay, because this approach contributes to definitive diagnosis. Especially for highly suspected iCCA cases with unresectable tumor, pretransplant biopsy is warranted. When iCCA is diagnosed definitively, pretransplant bridging treatment should be arranged next. But, if pretransplant biopsy is not feasible, favorable biology was referred when tumor lesion responses well to systemic neoadjuvant chemotherapy or/and locoregional therapy. Finally, LT is recommended as a viable modality for those responders. Probably, under these protocols, the therapeutic role of LT in iCCA cases will be optimized in the near future.

Briefly, LT can achieve relatively satisfactory outcomes for “*very early stage*” iCCA cases (with single lesion ≤2 cm), and the transplant indication is also expected to expand to “*locally advanced stage*” iCCA cases (without extrahepatic metastasis or macrovascular invasion) but responses well to neoadjuvant chemotherapy. However, the concept of “*locally advanced*” remains to be defined in the future, especially the combination of “*maximum size of largest lesion*”, “*number of lesions*”, with/without “*tumor differentiation*”. These narrative results above are generated from literature review, and therefore, further precise consensus based on prospective, large-scaled, and well-designed study is still warranted, in order to better guide the management of LT in iCCA patients.

## Conclusions

In summary, LT alone or combined with neoadjuvant chemotherapy, can achieve relatively satisfactory outcomes in well selected iCCA patients. Importantly, tumor burden alone is not the determinant of transplant outcomes in iCCA patients, whereas, tumor burden, along with tumor biology, and response to pretransplant down-staging strategy should be taken into consideration before assessing LT in iCCA patients. Given the scarcity of donor organ, and also the debate about LT in iCCA, consensus for LT for iCCA patients is still urgently warranted.

## Author Contributions

DS conceived the topic and wrote this manuscript. JD and GL revised the manuscript. All the authors contributed to this article and approved the final submitted version.

## Funding

This research was supported by the Department of Science and Technology of Jilin Province (No. 20200603001SF).

## Conflict of Interest

The authors declare that the research was conducted in the absence of any commercial or financial relationships that could be construed as a potential conflict of interest.

## Publisher’s Note

All claims expressed in this article are solely those of the authors and do not necessarily represent those of their affiliated organizations, or those of the publisher, the editors and the reviewers. Any product that may be evaluated in this article, or claim that may be made by its manufacturer, is not guaranteed or endorsed by the publisher.
